# Current Communication Practices Between Obstetrics and Gynecology Residency Applicants and Program Directors

**DOI:** 10.1001/jamanetworkopen.2022.38655

**Published:** 2022-10-26

**Authors:** Helen Kang Morgan, Abigail Ford Winkel, Karen George, Eric Strand, Erika Banks, Fiona Byrne, David Marzano, Maya M. Hammoud

**Affiliations:** 1 Department of Obstetrics and Gynecology, University of Michigan, Ann Arbor; 2 Department of Learning Health Sciences, University of Michigan, Ann Arbor; 3 Department of Obstetrics and Gynecology, New York University Grossman School of Medicine, New York City; 4 Department of Obstetrics and Gynecology, The Larner College of Medicine at The University of Vermont, Burlington; 5 Department of Obstetrics and Gynecology, Washington University School of Medicine in St Louis, St Louis, Missouri; 6 Department of Obstetrics and Gynecology, Albert Einstein College of Medicine/Montefiore Medical Center, Bronx, New York; 7 University College Dublin, Dublin, Ireland

## Abstract

**Importance:**

In order to equitably improve the residency application process, it is essential to understand the problems we need to address.

**Objective:**

To determine how obstetrics and gynecology (OBGYN) applicants and faculty communicate applicants’ interest to residency programs, and how program directors report being influenced by these communications.

**Design, Setting, and Participants:**

This survey study was conducted with email surveys of OBGYN application stakeholders in 2022. Included participants were OBGYN applicants, clerkship directors, and residency program directors in medical education associations’ email listservs.

**Exposures:**

Surveys sent by the American Association of Medical Colleges, Association of Professors of Gynecology and Obstetrics, and Council on Resident Education in Obstetrics and Gynecology.

**Main Outcomes and Measures:**

Whether applicants themselves, or faculty on their behalf, communicated to residency programs, and the influence program directors reported placing on these communications for their decision-making. Descriptive statistics and χ^2^ tests were used to analyze differences.

**Results:**

A total 726 of 2781 applicants (26.1%) responded to the survey and were included in analysis (79 of 249 [31.7%] clerkship directors; 200 of 280 [71.4%] program directors). The self-reported racial and ethnic demographics of the 726 applicant respondents were 86 Asian (11.8%), 54 Black (7.4%), 41 Latinx (5.6%), 1 Native Hawaiian or Pacific Islander (0.1%), 369 White (52.2%), 45 with multiple racial identities (6.2%), and 91 (21.5%) preferring not to answer. The majority of applicants (590 [82.9%]) sent communications at some point in the application process. Applicants who identified as White (336 [88.7%]) or Asian (75 [87.2%]) were more likely than those who identified as Black (40 [74.1%]) or Latinx (33 [80.5%]) to reach out to programs (*P* = .02). There were also differences in type of medical school, with 377 of 427 MD applicants (88.3%), 109 of 125 DO applicants (87.2%), and 67 of 87 International Medical Graduate applicants (77.7%) reporting sending communications (*P* = .02). Approximately one-third (254 applicants [35.7%]) had faculty reach out to programs on their behalf. White (152 [40.1%]) and Asian (37 [43.0%]) applicants were more likely to have faculty reach out compared with Black (6 [11.1%]) and Latinx (12 [29.3%]) applicants (*P* = .01). Program directors reported that preinterview communications from faculty they knew (64 [32.2%]) and other program directors (25 [12.6%]) strongly influenced their decisions, and otherwise rarely reported that communications strongly influenced their decisions.

**Conclusions and Relevance:**

The current state of communications may increase inequities in residency application processes; differences between faculty communications for applicants from different racial and ethnic backgrounds are particularly concerning given that program directors are more likely to weigh communications from faculty in their decision-making. A centralized, equitable means for applicants to signal their interest to programs is urgently needed.

## Key Points

**Question:**

Are certain types of applicants disadvantaged by the current state of communications between applicants and residency programs?

**Findings:**

In this survey study, most applicants reported that they (or faculty acting on their behalf) reported sending communications to residency program directors. There were notable differences between MD, DO, and IMG applicants, as well as between applicants with different racial and ethnic backgrounds, with White and Asian applicants having more than double the rate of faculty advocating to residency programs on applicants’ behalf compared with Black or Latinx applicants.

**Meaning:**

These results suggest that, because the current state of communications may increase inequities in application processes, a centralized means for applicants to convey their interest to residency programs is urgently needed.

## Teaser

This survey study estimates racial and ethnic disparities in rates at which applicants to obstetrics and gynecology residency programs contact residency program directors and assesses how directors report being influenced by these communications.

## Introduction

The staggering inefficiencies created by application inflation are occurring in the context of obstetrics and gynecology (OBGYN) becoming an increasingly competitive specialty. In the 2021-2022 application cycle, there were 2781 total OBGYN applicants registered through the American Association of Medical Colleges (AAMC) Electronic Residency Application Service (ERAS).^1^ Of these 2781 applicants, 2161 (77.7%) ultimately registered through the National Residency Match Program (NRMP) for an OBGYN residency position, with 1499 (69.4%) successfully matching into OBGYN.^2^ Different types of applicants had markedly different match rates, with 1106 of 1358 MD applicants (81.4%), 242 of 396 DO applicants (61.1%), and 151 of 407 International Medical Graduate (IMG) applicants (37.1%) successfully matched into OBGYN residencies.^2^

While many factors contribute to the increased competitiveness of the specialty, marked application inflation creates unique challenges for both applicants and residency programs.^3^ In the past, when applicants submitted 10 to 20 applications, the application itself was a signal of interest, but this is no longer the case, with each OBGYN applicant submitting an average of 72 applications.^1^ Programs now struggle to ascertain which applicants are genuinely interested in their program and use easy-to-filter metrics to decrease their pools of applications to review to manageable numbers.^4,5^ Applicants have recognized that in order to increase the chance that their application will be reviewed, they (or faculty acting on their behalf) may need to communicate with the residency program to signal their genuine interest.

The application process should be carefully examined to understand how to create meaningful interventions to address inequities that may create disadvantages for some applicants. The OBGYN specialty has a substantial number of residents from DO (15.6%) and IMG backgrounds (7.3%),^6^ and these applicants may receive varying degrees of mentoring and advocacy compared with MD applicants. At this time, it is unknown how many applicants and faculty are reaching out to programs and whether different groups of applicants are disadvantaged through this informal signaling. This information is especially important as specialties consider the implementation of formal centralized signaling mechanisms. Preference or program signaling was first implemented by otolaryngology in the 2020-2021 application cycle,^7^ and 16 specialties are now offering it in the 2023 application season.^8^ Applicants can send formal signals to residency programs around the time they submit their application through the ERAS system. The aim of this study was to assess how OBGYN applicants and faculty are currently communicating their interest to residency programs, and how program directors report being influenced by these communications.

## Methods

Surveys were created for OBGYN applicants, OBGYN clerkship directors, and OBGYN residency program directors by the leadership team of the Right Resident, Right Program, Ready Day One initiative led by the Association of Professors in Gynecology and Obstetrics (APGO) and funded by an American Medical Association Reimagining Residency grant. The team consisted of medical school and residency educators, medical students, and residents from osteopathic (for DO applicants) and allopathic (MD) medical schools in the US, as well as international medical schools (IMG). Content validity was established through a review of the literature and response process validity was established through piloting of the survey with stakeholder groups.

For the applicant and clerkship director surveys, questions asked respondents if, when, and how they communicated with residency programs (eAppendices 1 and 2 in the Supplement). The applicant survey additionally asked if faculty corresponded with residency programs on behalf of the applicant. Applicants provided demographic information including category of medical school (MD, DO, or IMG) and self-reported race and ethnicity using predefined and free-text options. Applicants were instructed to include all races and ethnicities that applied. For the purpose of analyzing the data, responses were included in the following categories: Asian American in Asian; African American in Black; and Hispanic in Latinx. Respondents who marked more than 1 were classified as having multiple racial identities. For the program director survey, questions asked respondents if they received correspondence from applicants and faculty and how these communications influenced their decision-making during the application process (eAppendix 3 in the Supplement). The surveys were sent electronically to OBGYN applicants as emails through the AAMC ERAS, to OBGYN clerkship directors through the APGO clerkship director listserv in early March 2022, and to program directors through the annual Council on Resident Education in Obstetrics and Gynecology (CREOG) Program Director survey in January 2022. An email reminder was sent 1 week after the survey release.

The applicant and clerkship director surveys were deemed exempt from the need for informed consent from participants by the University of Michigan institutional review board. Likewise, the program director survey was deemed exempt by the Health Media Lab independent review board. All surveys were deemed to carry no more than minimal risk to respondents since the responses were anonymously collected. These studies followed the American Association for Public Opinion Research (AAPOR) reporting guidelines.^9^

### Statistical Analysis

Descriptive statistics were created using Excel version 16.60 (Microsoft Corp). χ^2^ tests were used to analyze differences between applicant characteristics and communications with residency programs using SPSS version 25. The significance level was set to *P* < .05 and 2-sided tests were used for hypothesis testing.

## Results

Of the 2781 total OBGYN applicants registered through ERAS, 726 responded, for a response rate of 26.1%. Based on self-reported race and ethnicity, 54 respondents (7.4%) identified as Black, 86 (11.8%) as Asian, 41 (5.6%) as Latinx or Hispanic, 1 (0.1%) as Native Hawaiian or Pacific Islander, and 379 (52.2%) as White (Table 1). Of the 726 respondents, 712 (98.1%) included information about their medical school and how they corresponded with residency programs. The percentage of MD, DO, and IMG applicants among the respondents were similar to the proportions of these applicant groups both among those registered through ERAS and those who ultimately matched into OBGYN through the NRMP (Table 2).

**Table 1.** Self-reported Racial and Ethnic Demographics of OBGYN Applicants

Race or ethnicity	Survey respondents, No. (%) (n = 726)	All residents in ACGME-accredited OBGYN Programs, No. (%) (n = 5608)^a^
Asian	86 (11.8)	870 (15.5)
Black	54 (7.4)	489 (8.7)
Latinx	41 (5.6)	595 (10.6)
Native Hawaiian or Pacific Islander	1 (0.1)	2 (<0.01)
White	379 (52.2)	3501 (53.8)
Multiple racial identities	45 (6.2)	221 (3.9)
Other	12 (1.7)	NA
Prefer not to answer	17 (2.3)	NA
Did not answer	91 (12.5)	NA

Abbreviations: ACGME, Accreditation Council for Graduate Medical Education; NA, not applicable; OBGYN, obstetrics and gynecology.

^a^ As of December 2020.

**Table 2.** Medical School Demographics of OBGYN Applicants

Type of medical school	Applicants, No. (%)		
	Survey respondents (n = 712)	Registered in ERAS 2022 (n = 2781)	Registered in NRMP 2022 (n = 2161)
MD senior or graduate	427 (60.0)	1564 (56.2)	1358 (62.8)
DO senior or graduate	125 (17.6)	520 (18.7)	396 (18.3)
IMG senior or graduate (US and non-US schools)	87 (12.2)	697 (25.1)	240 (11.1)
Other	NA	NA	167 (7.7)
Did not answer	73 (10.3)	NA	NA

Abbreviations: ERAS, Electronic Residency Application Service; IMG, International Medical Graduate; NRMP, National Resident Matching Program; OBGYN, obstetrics and gynecology.

**Figure 1.**
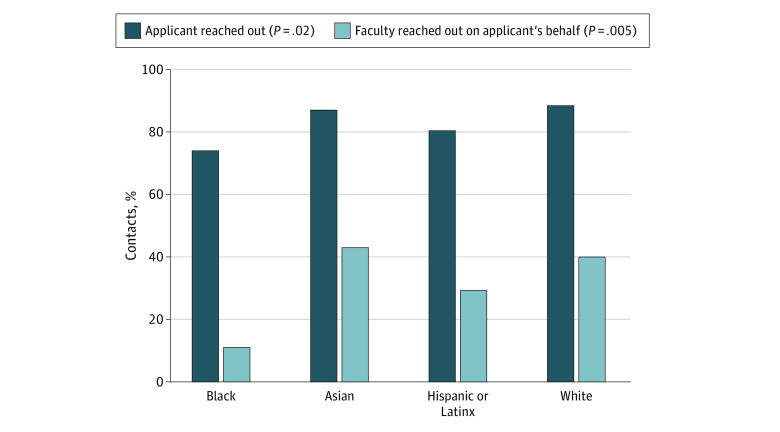
Racial and Ethnic Differences in Applicants and Faculty Reaching Out to OBGYN Residency Programs OBGYN indicates obstetrics and gynecology.

Most respondents (590 [82.9%]) sent communications signaling interest to residency programs at some point during the application and interview season. Different applicant groups had different rates of communication, with 377 MD applicants (88.3%), 109 DO applicants (87.2%), and 67 IMG applicants (77.7%) reporting that they reached out to programs (*P* = .02). In addition to personally reaching out themselves, more than one-third of all respondents (254 [35.8%]) had faculty contact programs on their behalf. Less than half (323 respondents [45.4%]) did not contact programs, 116 (16.3%) were not sure, and 19 (2.7%) did not answer this question. Notably, there were marked differences between groups based on type of medical school. Nearly half of MD applicants (189 [44.3%]) had faculty reach out, compared with 26 DO applicants (20.8%), and 20 IMG applicants (23.0%) (*P* < .001). There were also notable differences between self-identified racial or ethnic groups. Applicants identifying as White (336 [88.7%]) or Asian (75 [87.2%]) were more likely than applicants identifying as Black (40 [74.1%]) or Latinx (33 [80.5%]) to contact programs directly (*P* = .02). White and Asian applicants were also 2 to 3 times more likely to have faculty reach out to programs on their behalf (*P* = .01) (Figure 1).

Applicants’ methods of communication with residency programs were predominantly via email (578 respondents [81.2%]), with 101 respondents (14.2%) calling the program and 71 (10.0%) writing a physical letter. For faculty communication with programs, 197 respondents (27.7%) reported that faculty emailed, 83 (11.7%) that faculty called, and 5 (1.0%) that faculty wrote a physical letter to the residency program.

Many applicants reported reaching out to a substantial number of residency programs in an effort to secure an interview: 44 (6.6%) reached out to 1 program, 117 (17.5%) reached out to 2 or 3, 98 (14.6%) reached out to 4 or 5, 126 (18.8%) reached out to between 6 to 10, 86 (12.8%) reached out to between 11 to 20, and 83 (12.4%) reached out to more than 20 programs. Only 116 respondents (17.3%) did not reach out to any programs. Forty-two respondents (6.3%) did not answer this question.

After interviews were completed, respondents reached out to a fewer number of programs, with 209 (31.2%) reaching out to only 1 program, 228 (34.0%) reaching out to 2 or 3, 77 (11.5%) reaching out to 4 or 5, 43 (6.4%) reaching out to between 6 and 10, 12 (1.8%) reaching out to between 11 and 20, 7 (1.0%) reaching out to more than 20 programs, and 94 (14.0%) reaching out to no programs. Forty-two (6.3%) respondents did not answer this question.

**Figure 2.**
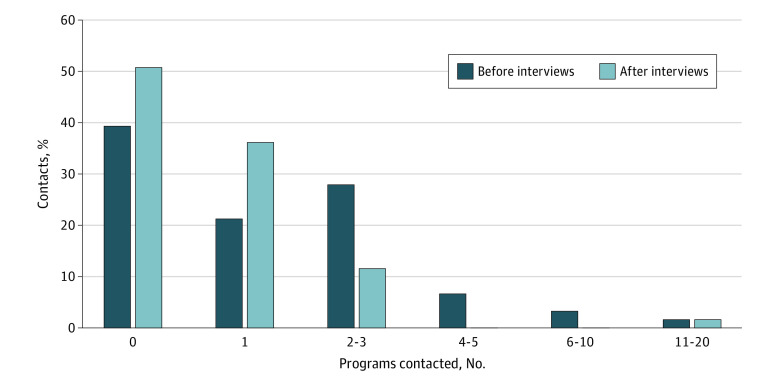
Percentage of Clerkship Directors Who Reached Out to Programs on Behalf of Their Students Before and After Interviews

Among clerkship directors, 79 of 249 responded, for a response rate of 31.7%. Of these, more than half (41 respondents [51.9%]) reached out to residency programs on behalf of their students during the application and interview process (Figure 2). Clerkship directors spent an average of 3.1 hours per student contacting programs.

**Figure 3.**
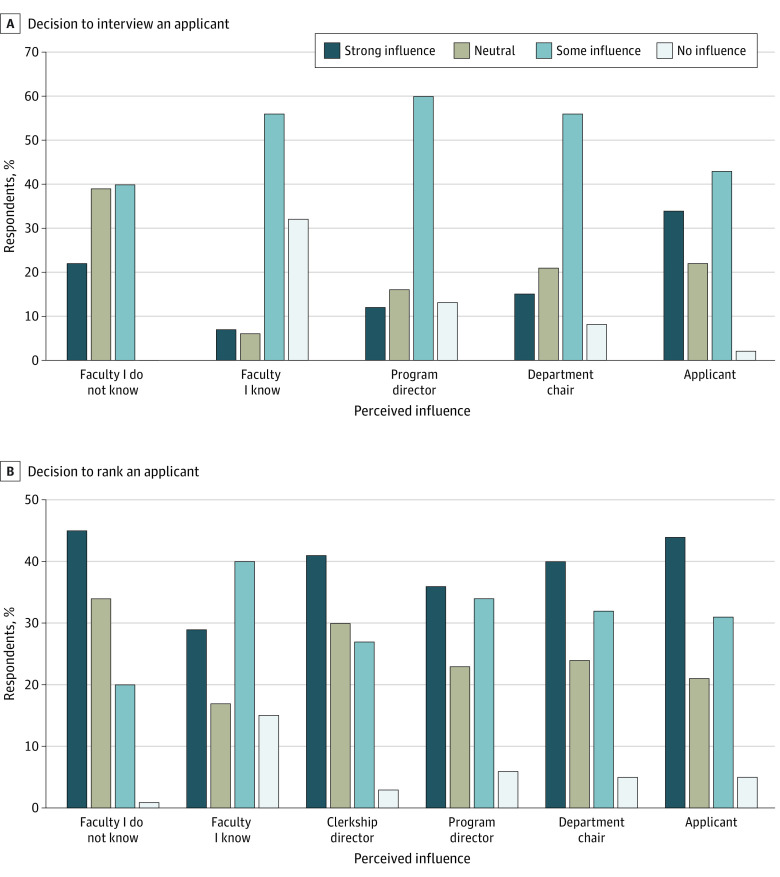
Program Directors’ Perceptions of the Influence of Email Communication on the Decisions to Interview and Rank an Applicant

Among program directors, 200 of 280 responded to the survey, for a response rate of 71.4%. Most respondents were not strongly influenced by any of the communications from different groups when making decisions about which applicants to interview. However, they were influenced most by communications from faculty that they knew, or from fellow program directors (Figure 3). Notably, 176 program directors (88.0%) reported that email from a faculty that they knew would influence or strongly influence their decision to interview an applicant. In addition, 110 program directors (56.0%) reported that email communication from the applicant had either no influence or neutral influence on their decision to offer an interview to the applicant and 130 (65.0%) reported that email communication from applicants either had minimal or no influence on their decision-making about ranking an applicant (Figure 3).

## Discussion

Our study highlights the many inequities in the current residency application processes. Applicants and faculty are already trying to send many signals to residency program directors. The notable differences between MD, DO, and IMG applicants, as well as applicants from different racial and ethnic backgrounds, are alarming. The fact that White and Asian applicants had more than double the rate of faculty advocating to residency programs on their behalf is especially notable given that program directors reported being more influenced by communications from faculty and fellow program directors. While clerkship directors are also spending considerable time reaching out on behalf of their students, these communications may not be helpful if they do not know the program director—highlighting yet another potential inequity for applicants with faculty who may be less connected to other medical educators in OBGYN. Finally, the amount of effort applicants spend contacting programs with questionable benefit is remarkable, highlighting an opportunity cost that could have been spent on service, scholarship, and preparing for residency.

An equitable application process should empower applicants to have fair access to transparent selection systems. Our findings reveal that this is not the case. The proverbial “smoke-filled back rooms” have been replaced by emails and calls from colleagues that can influence program directors’ decision-making. DO, IMG, and applicants from racial and ethnic backgrounds underrepresented in medicine might not have equal access to faculty to advocate on their behalf. This varying access could be due to medical school structure, varying resources, or the lack of faculty mentorship for individuals from underrepresented backgrounds. This has been further exacerbated during the COVID-19 pandemic as visiting rotations have been discontinued or have reduced availability,^10^ further limiting access for these applicant groups to mentors and faculty outside their school who could reach out to residency programs on their behalf. Applicants from majority backgrounds may feel more entitled or comfortable asking faculty to send communications for them. It is important to highlight how these inequities can work synergistically to disadvantage applicants from already marginalized backgrounds. Because of application inflation, program directors use filters such as clerkship grades, Alpha Omega Alpha medical honor society status, and standardized test scores.^4,11^ These metrics are known to be vulnerable to racial and ethnic bias and to favor White applicants.^12,13^ Our findings show that White and Asian applicants are further advantaged at this point by having faculty who reach out to program directors, which may subsequently enable their applications to be more holistically reviewed. The percentage of Black OBGYN residents has been decreasing in the past decade,^14,15^ and these additive inequities in the current processes are likely one of the many contributors to this troubling decline. Our study demonstrates how existing systems are further propagating societal inequities in the application process and highlights the need for a centralized, equitable way for applicants to signal their interest in programs. Instead of needing to rely on faculty relationships that are out of an applicant’s control, a formal program signaling system should work to level this uneven playing field.

The inequitable faculty advocacy for different types of applicants is especially relevant given that the first 2 years of the otolaryngology preference signaling experience demonstrated that the impact of the signals was greatest for applicants who struggle to receive many interview offers.^16^ Of the 16 specialties that are implementing program signaling in the 2022-2023 application, most (14 of 16) allow applicants to send up to 5 or 10 signals. In OBGYN, applicants will be able to send out a total of 18 signals (3 highest priority and 15 high priority) to programs in the 2023 application cycle. Our study suggests that a higher number of signals may be beneficial to applicants given that more than half of our survey respondents reached out to more than 4 programs. However, it is challenging to infer whether these applicants were reaching out to these large numbers of programs because of a genuine interest in the program, or whether they were simply hoping to increase their chances of getting residency interviews. It is also important to note that a substantial number of applicants are registered through ERAS who ultimately do not count as active applicants for the Match through NRMP. What happens to these applicants and how many of them dual-apply into a second specialty is unclear at this time. Increased transparency and sharing of data available through ERAS and NRMP will be essential as change measures such as program signaling are implemented and evaluated.

Our study highlights that applicants are also trying to communicate their interest in residency programs after interviews have been completed. While it is notable that applicants are sending communications to fewer numbers of programs after interviews, this data questions whether these communications should continue at all given how little value program directors seem to place on these emails, as well as the inequity considerations. This is consistent with previously published work that has questioned the value of these postinterview communications.^17^ The purpose of this communication is notably different from that of the preinterview communication since the applicant has presumably been able to express their interest in the program during the interview day.

### Limitations

This study had several limitations. The response rates were lower for applicants and clerkship directors, but we were still able to capture representative samples from stakeholders. The response rate was higher for program directors because the survey questions were included in an annual survey of program directors.

We did not ask questions about what content was included in communications from applicants, faculty, and clerkship directors. However, our study highlights that, in the midst of application inflation, program signaling is needed in order for applicants to equitably communicate their interest to residency programs.

Finally, it is important to note that program signaling does not resolve the underlying problem of application inflation. If anything, it might increase the number of applications submitted per applicant.^1^ While it is tempting to treat a symptom of the bigger problem when working to achieve a more equitable residency application process, there remain a number of inequities when considering the burden of time and cost on individual applicants. Program signaling, especially in those specialties using a high number of signals, may provide more clarity on the feasibility of potential application capping in the future.

## Conclusions

The myriad of consequences created by application inflation has led to a worsening of inequities in the residency application process. This study highlights the need for central program signaling, especially given otolaryngology data that suggests that centralized signaling may be the most beneficial for applicants who struggle to receive many interview offers.

## Notes

Supplement. **eAppendix 1.** Applicant Survey **eAppendix 2.** Clerkship Director Survey **eAppendix 3.** Program Director Survey

## References

[1] Association of American Medical Colleges. ERAS Statistics. Accessed June 20, 2022. https://www.aamc.org/data-reports/interactive-data/eras-statistics-data

[2] National Resident Matching Program. Main Residency Match Data and Reports. Accessed June 20, 2022. https://www.nrmp.org/match-data-analytics/residency-data-reports/

[3] Gruppuso PA, Adashi EY. Residency placement fever: is it time for a reevaluation? Acad Med. 2017;92(7):923-926. doi:10.1097/ACM.0000000000001468 27805954 PMC5411322

[4] Garber AM, Kwan B, Williams CM, . Use of filters for residency application review: results from the internal medicine in-training examination program director survey. J Grad Med Educ. 2019;11(6):704-707. doi:10.4300/JGME-D-19-00345.1 31871573 PMC6919169

[5] Pereira AG, Chelminski PR, Chheda SG, ; Medical Student to Resident Interface Committee Workgroup on the Interview Season. Application inflation for internal medicine applicants in the match: drivers, consequences, and potential solutions. Am J Med. 2016;129(8):885-891. doi:10.1016/j.amjmed.2016.04.001 27154785

[6] Brotherton SE, Etzel SI. Graduate medical education, 2020-2021. JAMA. 2021;326(11):1088-1110. doi:10.1001/jama.2021.13501 34546319

[7] Pletcher SD, Chang CWD, Thorne MC, Malekzadeh S. The otolaryngology residency program preference signaling experience. Acad Med. 2022;97(5):664-668. doi:10.1097/ACM.0000000000004441 34618735 PMC9028299

[8] Association of American Medical Colleges. Supplemental ERAS application. Accessed June 20, 2022. https://students-residents.aamc.org/applying-residencies-eras/supplemental-eras-application

[9] American Association for Public Opinion Research. Standard Definitions. Accessed September 2, 2022. https://www.aapor.org/Publications-Media/AAPOR-Journals/Standard-Definitions.aspx

[10] Hammoud MM, Standiford TC, Carmody JB. The 2020-2021 residency application cycle: lessons learned and lingering problems. JAMA. 2021;325(22):2249-2250. doi:10.1001/jama.2021.5708 34019077

[11] National Resident Matching Program. Results of the 2021 NRMP Program Director Survey. Accessed June 20, 2022. https://www.nrmp.org/wp-content/uploads/2021/11/2021-PD-Survey-Report-for-WWW.pdf

[12] Low D, Pollack SW, Liao ZC, . Racial/ethnic disparities in clinical grading in medical school. Teach Learn Med. 2019;31(5):487-496. doi:10.1080/10401334.2019.1597724 31032666

[13] Boatright D, Ross D, O’Connor P, Moore E, Nunez-Smith M. Racial disparities in medical student membership in the Alpha Omega Alpha Honor Society. JAMA Intern Med. 2017;177(5):659-665. doi:10.1001/jamainternmed.2016.9623 28264091 PMC5818775

[14] López CL, Wilson MD, Hou MY, Chen MJ. Racial and ethnic diversity among obstetrics and gynecology, surgical, and nonsurgical residents in the US from 2014 to 2019. JAMA Netw Open. 2021;4(5):e219219. doi:10.1001/jamanetworkopen.2021.9219 34009352 PMC8134986

[15] Morgan HK, Winkel AF, Banks E, . Promoting diversity, equity, and inclusion in the selection of obstetrician-gynecologists. Obstet Gynecol. 2021;138(2):272-277. doi:10.1097/AOG.0000000000004469 34237768

[16] Chang CWD, Thorne MC, Malekzadeh S, Pletcher SD. Two-year interview and match outcomes of otolaryngology preference signaling. Otolaryngol Head Neck Surg. Published online August 30, 2022. 1945998221121312:1945998221121312. doi:10.1177/01945998221121312 36040808

[17] Grimm LJ, Avery CS, Maxfield CM. Residency postinterview communications: more harm than good? J Grad Med Educ. 2016;8(1):7-9. doi:10.4300/JGME-D-15-00062.1 26913094 PMC4763386

