# Errors in Abstract and Figure

**DOI:** 10.1001/jamanetworkopen.2022.52965

**Published:** 2023-01-12

In the Original Investigation titled “Current Communication Practices Between Obstetrics and Gynecology Residency Applicants and Program Directors,”^1^ published October 26, 2022, there was an error in the abstract and in a figure. The total of applicants not reporting their race was fixed to “91 (12.5%) did not indicate race and 17 (2.3%) preferred not to answer.” An error in the color coding for Figure 3 has also been corrected. This article has been corrected.^1^
