# Supplementary material for: Current Communication Practices Between Obstetrics and Gynecology Residency Applicants and Program Directors
Source: JAMA Netw Open. 2022 Oct 26;5(10):e2238655. doi: 10.1001/jamanetworkopen.2022.38655 (PMC9606842; doi:10.1001/jamanetworkopen.2022.38655)
Supplement: Supplement. — eAppendix 1. Applicant Survey eAppendix 2. Clerkship Director Survey eAppendix 3. Program Director Survey [file jamanetwopen-e2238655-s001.pdf]

Supplemental Online Content

Morgan HK, Winkel AF, George K, et al. Current communication practices between obstetrics and gynecology residency applicants and program directors. *JAMA Netw Open.* 2022;5(10):e2238655. doi:10.1001/jamanetworkopen.2022.38655

eAppendix 1. Applicant Survey

eAppendix 2. Clerkship Director Survey

eAppendix 3. Program Director Survey

This supplemental material has been provided by the authors to give readers additional information about their work.

© 2022 Morgan HK et al. *JAMA Network Open.*

eAppendix 1. Applicant Survey

Applicant survey 2022:

The following questions pertain to the residency application and interview processes in obstetrics and gynecology (OBGYN). Please try to answer all questions to the best of your ability as these are intended to evaluate changes made during the 2021-2022 application cycle as well as for future initiatives moving forward. Thank you and we value your input.

Section 1: Application Preparation and Standardized Letter of Evaluations

1. When did you begin working on your ERAS application?

a) June 2021
b) July 2021
c) August 2021
d) September 2021
e) Other_____

2. Approximately how many hours do you estimate that you devoted to your ERAS application?

a) 0-10 hours
b) 11-20 hours
c) 21-30 hours
d) 31-40 hours
e) 41-50 hours
f) >50 hours

3. Were you aware of the Standardized Letter of Evaluation (SLOE) pilot? Yes/No

*If No -> Then move on to question 4.

*If Yes -> 3a. Did you include a SLOE in your application package? Yes/No

*If Yes -> Then move on to question 4.

*If No-> 3b. Why did you not include a SLOE in your application package (check all that apply)

a. I did not have someone who could complete a SLOE for me
b. I chose not to because it was a pilot this year
c. I was advised not to include a SLOE because it was a pilot this year
d. I was worried that it would have a negative impact on my application package
e. Other______

4. Did you complete an away rotation prior to submission of your ERAS application? Yes/no

*If No-> Move on to question 5

*If Yes-> 4a. What was the **primary purpose** of your away rotation(s)? (please select the one most important purpose)

© 2022 Morgan HK et al. *JAMA Network Open.*

a) Opportunity to signal my interest in a program
b) Opportunity to demonstrate how I might function in a program
c) Opportunity for a clinical experience that I may not have available at my institution
d) Other ______

4b. Did you receive an interview offer from your away rotation program? Yes/no

Section 2: Correspondence with Residency Programs

5. Did you, or a faculty member on your behalf, reach out to programs during the application and interview season? yes/no

*If no-> Move on to question 6

*If Yes-> 5a. Please complete this grid indicating how your reached out to residency programs (check all that apply)

	Before the interview offer release date	After the October 18 interview offer release date	Immediately after I completed my interview at that program	At the time that I submitted my rank list
I emailed the program				
I called the program				
I sent regular mail				
A faculty sent an email on my behalf				
A faculty made a phone call on my behalf				
A faculty sent a regular mail on my behalf				

5b. How many programs did you send a correspondence to, with the hopes of procuring a residency interview?

a. 0
b. 1
c. 2-3
d. 4-5
e. 6-10
f. 11-20
g. >20

© 2022 Morgan HK et al. *JAMA Network Open.*

5c. How many programs did a faculty members reach out to on your behalf, with the hopes of helping you to procure a residency interview?

a. 0
b. 1
c. 2-3
d. 4-5
e. 6-10
f. 11-20
g. >20

6. How many programs did you send some correspondence to, expressing high interest in the program, after you completed an interview? (personally or through a faculty member)

a. 0
b. 1
c. 2-3
d. 4-5
e. 6-10
f. 11-20
g. >20

Section 3: Second look

7. Did you perform any in-person "second look" visits? Yes/No

*If no -> Go to question 8

*If yes-> 7a. How many in-person "second look" visits did you perform?

a. 1
b. 2
c. 3
d. 4
e. 5 or more

7b. Who did you meet with from the institution during the "second look" visit?

a. I did not meet with anyone from the institution
b. Residency program director or associate residency program director
c. Program Manager
d. Residents in the program
e. OBGYN faculty
f. Other non-OBGYN staff or faculty from the institution

7c. Please rank the following reasons for your "second look" visit? (1)=most important to (4)=least important

__Opportunity to signal my interest in a residency program

__Opportunity to visit the surrounding area

___Since the visit was offered by the program, I was concerned of potential negative repercussions if I did not attend

____Other_______

8. Why did you choose not to perform any "second look" visits? (check all that apply)

    a. They were not offered by my programs

    b. Unable to travel secondary to financial limitations

    c. Unable to travel secondary to logistical limitations

    3. I did not feel a second look would impact my ranking of residency programs

    d. Other______

Please fill in the following. Please enter whole numbers- enter 0 if none. If you cannot recall exactly use your best estimate.

9a. Number of applications you submitted ____

9b. Number of interview invitations you received

____

9c. Number of interviews you completed ____

9d. Number of interview invitations you received on October 19, 2021

____

9e. Number of interview invitations which indicated you had 48 hours to respond to the interview offer

____

9f. Number of programs that offered you an interview (that was not specified as a wait list) and did not have an interview spot ____

10. How many specialties did you apply to in addition to OBGYN (enter 0 if none)

____

11. What was your first specialty choice?

▼ OBGYN ... Other

© 2022 Morgan HK et al. *JAMA Network Open.*

Display This Question:

If = Other

11a. Please enter the name of your first choice specialty

____

Section 4: Future Initiatives

12. Please rank the following interview options which you believe programs should consider in the future from most desirable (1) to least desirable (4). Please assume that in-person options have no restrictions (as in pre-COVID times).

______ All interviews are conducted exclusively virtually
______ All interviews are conducted virtually with open house in person visit option
______ All interviews are conducted in person
______ Applicants have the option of virtual or in person interview

13. OBGYN will be adding **two tiers** of preference signaling in future application cycles. Applicants would be able to signal three residency programs with their highest three preferences (Gold signals), as well as their next top 15 preferences (Silver signals) at the time of the submission of the ERAS application. How do you believe this would have impacted your residency application process?
    (a) This would have been very helpful to me
    (b) This would have been helpful to me
    (c) Neutral
    (d) This would have been unhelpful for me
    (e) This would have been very unhelpful for me
    (f) Please share any additional thoughts or comments pertaining to the proposed preference signaling idea_________

The following questions pertain to the addition of situational judgement tests such as the ALTUS suite of assessments. In 2021-2022, nine OBGYN programs used the ALTUS suite of assessments in their residency application process.

14. By completing the ALTUS suite of assessments, the residency program learned something novel that was not in my ERAS package
    a. Strongly disagree
    b. Disagree
    c. Neutral
    d. Agree
    e. Strongly Agree
    f. I did not complete the ALTUS suite of assessments

15. If additional cost were not a factor, Situational Judgment Tests (eg. Altus) should be a component of the residency application
    a. Strongly disagree
    b. Disagree
    c. Neutral
    d. Agree
    e. Strongly Agree

Please provide any additional information that you would like to share pertaining to the ALTUS or other situational judgment tests ________________

Display This Question:

If If Please fill in the following. Please enter whole numbers- enter 0 if none. If you cannot recall e... How many specialties did you apply to in addition to OBGYN (enter 0 if none) Is Not Equal to 0

16. Are you couple's matching?
    ii) Yes
    iii) No

---

17. Please check your USMLE Step 1 score or COMLEX 1 score
    (1) USMLE < 200 and COMLEX <487
    (2) USMLE 200-220 or COMLEX 488-575
    (3) USMLE 221-240 or COMLEX 576-660
    (4) USMLE 241-260 or COMLEX 661-742
    (5) USMLE >260 or COMLEX >742

© 2022 Morgan HK et al. *JAMA Network Open.*

18. Please check the status that applies to you when you submitted your application in the 2020-2021 residency application cycle

a) US Senior-MD

b) US Alumni-MD

c) US Senior-DO

d) US Alumni-DO

e) IMG Senior (final year)-US Citizen

f) IMG Alumni- US Citizen

g) IMG Senior (final year)-Non US Citizen

h) IMG Alumni-Non US Citizen

19. What is your racial/ethnic identity (select all that apply)?
    a. African American or Black
    b. American Indian or Alaska Native
    c. Asian or Asian American
    d. Latin(x) or Hispanic
    e. Native Hawaiian or Pacific Islander
    f. White
    g. Prefer not to Answer
    h. Self-describe:____________

---

20. Please share any additional comments/concerns/suggestions about the residency application and interview cycle.

____

____

____

____

____

© 2022 Morgan HK et al. *JAMA Network Open.*

eAppendix 2. Clerkship Director Survey

Clerkship Director survey 2022:

The following questions pertain to the residency application and interview processes in obstetrics and gynecology (OBGYN). Please try to answer all questions to the best of your ability as these are intended to evaluate changes made during the 2021-2022 application cycle as well as for future initiatives moving forward. Thank you and we value your input.

Section 1: Application Preparation and Standardized Letter of Evaluations

1. Were you aware of the OBGYN Standardized Letter of Evaluation (SLOE) pilot? Yes/No

*If No -> Then move on to question 2.

*If Yes -> 1a. Did you encourage applicants to include a SLOE in their application package? Yes/No

*If Yes -> Then move on to question 2

*If No-> 1b. Why did you not encourage applicants to include a SLOE in their application package? (check all that apply)

a. Applicants did not have someone who could complete a SLOE for them
b. This was a pilot this year
c. Worried that the SLOE would have a negative impact on their application package
d. Other______

2. How many students at your school applied to OBGYN residency in the 2021-2022 season? (enter a whole number, enter 0 if none)

____

3. How many students at your school applying to OBGYN residency did not match in the 2020-2021 season? (enter a whole number, enter 0 if none)

____

Section 2: Correspondence with Residency Programs

4. Did you reach out to programs on behalf of your students during the application and interview season? yes/no

*If no-> Move on to question 5

*If Yes-> 4b. Approximately how many programs per student did you reach out to in order to procure a residency interview?

a. 0

© 2022 Morgan HK et al. *JAMA Network Open.*

b. 1
c. 2-3
d. 4-5
e. 6-10
f. 11-20
g. >20

4c. Approximately how many programs per student did you reach out to, in order to express their high interest in a program, after they had completed an interview?

a. 0
b. 1
c. 2-3
d. 4-5
e. 6-10
f. 11-20
g. >20

4d. Approximately how many hours did you spend reaching out to residency programs on behalf of your students? ______________________ hours

Section 4: Future Initiatives

5. Please rank the following interview options which you believe programs should consider in the future from most desirable (1) to least desirable (4). Please assume that in-person options have no restrictions (as in pre-COVID times).

______ All interviews are conducted exclusively virtually
______ All interviews are conducted virtually with open house in person visit option
______ All interviews are conducted in person
______ Applicants have the option of virtual or in person interview

6. OBGYN will be adding **two tiers** of preference signaling in future application cycles. Applicants would be able to signal three residency programs with their highest three preferences (Gold signals), as well as their next top 15 preferences (Silver signals) at the time of the submission of the ERAS application. How do you believe that this will impact your learners' residency application process?
   (a) This will be very helpful for applicants
   (b) This will be helpful for applicants
   (c) Neutral
   (d) This will not be helpful for applicants
   (e) This will be very unhelpful for applicants
   (f) Please share any additional thoughts, comments or concerns pertaining to the proposed preference signaling idea_________

© 2022 Morgan HK et al. *JAMA Network Open.*

The following questions pertain to the addition of situational judgement tests such as the ALTUS suite of assessments. In 2021-2022, nine OBGYN programs used the ALTUS suite of assessments in their residency application process.

7. If additional cost were not a factor, Situational Judgment Tests (eg. Altus) should be a component of the residency application
    a. Strongly disagree
    b. Disagree
    c. Neutral
    d. Agree
    e. Strongly Agree

Please provide any additional information that you would like to share pertaining to the ALTUS or other situational judgment tests ______________

---

8. Please share any additional comments/concerns/suggestions about the residency application and interview cycle.

____

____

____

____

____

© 2022 Morgan HK et al. *JAMA Network Open.*

eAppendix 3. Program Director Survey

2022 CREOG Program Director Survey

Informed Consent

Please complete the following survey to share your thoughts about the transition into and out of residency and resident wellness. The information will be used to inform development of resources to improve the transition into and out of residency, and future research. This survey should take approximately 10-15 minutes to complete. Your participation is voluntary. Some of the questions are personal but help us to better understand your stressors. You may skip any questions that you do not feel comfortable answering and may withdraw from the survey at any point. **The survey responses are never associated with your personal identity or your program. All data are de-identified and aggregated for analyses and reporting by CREOG leadership.**

If you have any questions, comments, or concerns after participating in this survey please be in touch with me.

Karen George, MD, MPH

CREOG Chair

kageorge@mfa.gwu.edu

If you wish to be provided a copy of this consent, please contact the CREOG office at CREOG@ACOG.org

I consent to participating in the CREOG survey

- Yes
- No

Demographics Questions

1. Please indicate the geographic region of your residency
   a) New England (ME, NH, VT, MA, RI, CT)
   b) Middle Atlantic (NJ, NY, PA)
   c) East North Central (OH, MI, IN, IL, WI)
   d) West North Central (MN, KS, NE, IA, MO, ND, SD)
   e) South Atlantic (DE, DC, FL, GA, MD, NC, SC, VA, WV)
   f) East South Central (AL, KY, MS, TN)
   g) West South Central (AR, LA, OK, TX)
   h) Mountain (AZ, CO, ID, UT, NM, NV, MT, WY)

© 2022 Morgan HK et al. *JAMA Network Open.*

i) Pacific (CA, OR, WA, HI, AZ)

2. Please indicate the size of your residency program
   a) ≤ 4 residents per PGY year
   b) 5-7 residents per PGY year
   c) 8-10 residents PGY per year
   d) 11 or more residents per PGY year

3. What proportion of your current residents are from U.S. allopathic medical schools?
   a. 100%
   b. 75-99%
   c. 50-74%
   d. 25-49%
   e. 1-24%
   f. 0%

4. What proportion of your current residents are from U.S. osteopathic medical schools?
   a. 100%
   b. 75-99%
   c. 50-74%
   d. 25-49%
   e. 1-24%
   f. 0%

5. What proportion of your current residents are from international medical schools?
   a. 100%
   b. 75-99%
   c. 50-74%
   d. 25-49%
   e. 1-24%
   f. 0%

6. Which of the following terms best describes your program
   a) Community-based
   b) University-based
   c) Combination community/university based
   d) Military-based

7. How would you describe yourself? (choose all that apply):
   a) American Indian or Alaska Native
   b) Asian
   c) Black or African American
   d) Native Hawaiian or Other Pacific Islander
   e) White
   f) Hispanic, Latinx, or Spanish origin
   g) An identity not listed

© 2022 Morgan HK et al. *JAMA Network Open.*

h) Prefer not to answer

5. To which gender identity do you identify most:

a) Female
b) Male
c) Nonbinary
d) An identity not listed
e) Prefer not to answer

6. What is your age category?

a) 30-39
b) 40-49
c) 50-59
d) 60-69
e) 70 or over
f) Prefer not to answer

7. How long have you had your current position as program director

a) 2 years or less
b) 3-5 years
c) 6-10 years
d) More than 10 years

8. What is your current level of satisfaction with your position as program director?

a) Very satisfied
b) Satisfied
c) Neutral
d) Unsatisfied
e) Very unsatisfied

Transition to Residency

9. Does your institution offer “away rotations” for medical students from other institutions (please do not consider required 3rd year clerkships as away rotations)?

a) No
b) Yes

© 2022 Morgan HK et al. *JAMA Network Open.*

IF YES:

What is primary purpose of the away rotation(s)?

e) Opportunity for applicants to signal their interest in my program
f) Opportunity for applicants to demonstrate how they might function in my program
g) As a way to show off my program to students to recruit them
h) To offer a clinical experience to students who may not have a similar opportunity at their institution
i) Other reason_(allow write in)_______

Do you, as the PD, screen students before they are accepted for an away rotation?

a) Yes—students are only accepted if I approve
b) There is someone in Ob/Gyn that screens them and we have agreed upon criterion
c) There is someone in Ob/Gyn that screens them and I am not aware of criterion they use
d) No—students are only screened by the medical school or institutional educational officer to determine eligibility

Are the students who complete away rotations in your institution guaranteed an interview for your residency program?

a) Yes
b) No

Prior to covid pandemic restrictions, approximately what percentage of your residents completed an away rotation at your institution?

a) none
b) 1-25%
c) 21-50%
d) 51-75
e) >75%
f) Not sure

10. Approximately what percentage of applicants to your program submitted at least 1 Standardized Letter of Evaluation (SLoE) this past application cycle?

a) >75%
b) 50-74%
c) 25-49%
d) <25%
e) Not sure

11. How helpful were the **S**tandardized **L**etters **of Evaluation** (SLoE) in the application review process compared to the traditional letters of **recommendation**

a. Much more helpful
b. Somewhat more helpful

© 2022 Morgan HK et al. *JAMA Network Open.*

c. Neutral
d. Not as helpful
e. Too soon to tell

12. Assuming that interviews continue virtually, how do you feel about the amount of time allocated to review applications between the release of applications on 10/1 and the standardized interview release date on 10/19 as suggested by current specialty guidelines:

a. Not enough, would like more time to review applications before interview decision deadline
b. It was the right amount of time to review all the applications and make a decision on which candidates to interview
c. It is too much time and would like it to be a shorter interval

13. If Ob/Gyn recommended a supplementary application (one or at most 2 short questions completed by applicants written by your program before application review), do you think that would help your review process?

a. Yes
b. No
c. Not sure

14. Communication with stakeholders during interview season

Pre-interview communication

How much do the following communications influence your **decision to interview** an applicant	Does not influence	Neutral	Has some influence	Strongly influences
Email from an applicant				
Email from a Department Chair				
Email from a Program Director				
Email from a Clerkship Director				
Email from a Faculty I know				
Email from a Faculty I don't know				

Post-interview communication

© 2022 Morgan HK et al. *JAMA Network Open.*

How much do the following communications influence your **decision to rank** an applicant	Does not influence	Neutral	Has some influence	Strongly influences
Email from an applicant				
Email from a Department Chair				
Email from a Program Director				
Email from a Clerkship Director				
Email from a Faculty I know				
Email from a Faculty I don't know				

15. Please indicate the extent to which you agree with the following statements:

	**Strongly Disagree**	**Disagree**	**Neutral**	**Agree**	**Strongly Agree**
I use holistic review methods (balanced consideration to applicants' academic metrics, experiences, and attributes) as opposed to "threshold screening" to select candidates for interviews					
Now that I know my PGY-1 class, I feel that I was able to sufficiently gauge their attributes and challenges in a virtual interview setting					
In general, I feel that my new interns are well prepared for residency when they arrive to my program					
A standardized specialty-wide post-match curriculum would be helpful to prepare matched students for residency					
A standardized specialty-wide assessment tool to assess interns at the start of residency would be helpful					

16. After the match and prior to the start of residency, do you assign any learning tasks to your matched interns (not including GME assigned e-learning modules or tasks).

a) No
b) Yes, learning tasks suggested or recommended
c) Yes, learning tasks are required or mandatory

© 2022 Morgan HK et al. *JAMA Network Open.*

15A If yes, please select which tasks you assign (select all that apply)

1. CREOG post-match curriculum
2. Other readings (write in)
3. Other types of learning modules such as videos or podcasts (write in)
4. Other tasks (write in)

17. When do your interns begin typically begin their first rotation in your residency? i.e. begin patient care activities

a) Before 6/25
b) Between 6/25 and 7/1
c) July 1 or after

18. How much time before the first day on rotation do you interns spend in orientation and/or 'bootcamp'?

a. 1-7 days
b. 8-14 days
c. 15-21 days
d. >21 days

19. At the start of residency, do you perform a structured assessment of knowledge, skills, and attributes of the incoming class

a) No
b) Yes

16A If yes, please indicate methods used for assessment

1. Incoming residents provide a self-assessment of their strengths and weaknesses
2. APGO Preparation for Residency Knowledge Assessment Tool (uWISE)
3. Objective Standardized Clinical Evaluations
4. Objective Surgical Skill Assessment
5. Any other objective knowledge assessment
6. Other (write in)

20. In your opinion, approximately what percentage of your newest cohort of residents were satisfactorily prepared in medical school to begin internship?

a) 100%
b) 75%
c) 50%
d) 25%
e) 0%

© 2022 Morgan HK et al. *JAMA Network Open.*

21. Please indicate the areas where you feel entering residents are not prepared: (select all that apply)

a. Technical skills – basic suturing skills
b. Technical skills – ultrasound
c. Medical knowledge – routine inpatient issues
d. Medical knowledge – routine outpatient issues
e. Making an assessment or a plan
f. Communication with inter-professional team members (e.g., nurses, consultants, social workers, etc.)
g. Communication with patients and their families
h. Communication with difficult patients
i. Presentations on rounds
j. Documentation skills
k. How to obtain informed consent
l. How to give/receive a sign out (e.g., handoff, handover)
m. Skills for covering other services (cross cover) on call
n. Recognizing and preventing burnout
o. Evidence-based medicine
p. Time management
q. Outpatient clinic skills
r. End-of-life care
s. Cost-effective care
t. Professionalism
u. Other (write in)
v. My new interns were adequately prepared in all of these areas

Resident Health and Wellbeing

22. In your program, what is your perception of the importance placed on resident wellness (defined as the active pursuit of good health of all team members) in relation to other residency requirements?

a) A strong priority in my program
b) Somewhat a priority
c) Not a priority
d) Not sure/don't know

**22. During this academic year (since July 2021) are you aware of any of the following symptoms or behaviors in any of your residents:**		
	No	Yes
**depression or anxiety?**	☐	☐
**symptoms of burnout?**	☐	☐

© 2022 Morgan HK et al. *JAMA Network Open.*

**binge drinking?**	☐	☐
**use of illegal or illicit prescription or non-prescription substances?**	☐	☐
**thoughts about leaving your residency program?**	☐	☐
**thoughts of suicide?**	☐	☐

Transition to Fellowship and Practice

23. Do you allow for protected study time for the qualifying exam at the end of PGY-4?

  a. No
  b. Yes
     If yes, how much time?

     i. 7 or fewer days
     ii. Between 1-2 weeks
     iii. >2 weeks

24. What is the typical last day of patient care assignments for PGY-4 residents in your program?

  a. June 30
  b. Between 6/22 and 6/29
  c. Between 6/14 and 6/21
  d. Before June 14

25. Approximately what percentage of your residents apply for fellowship?

  a. 0-25%
  b. 26-50%
  c. 51-75%
  d. 76-100%

26. As you think about the transition to fellowship or practice for your residents, which of the following options is your preferred choice for the timing of their qualifying exam (written boards):

  a. Written boards on the last Monday of June
  b. Written boards first week in July
  c. Written boards second week of July
  d. Another option not listed, specify________

27. Considering your preferred timing for the written boards (question 14 above) which of the following do you think is the best choice for the first day of fellowship?

  a. July 1
  b. July 7th
  c. July 15th,
  d. August 1

© 2022 Morgan HK et al. *JAMA Network Open.*

28. How do you feel about staggering fellowship interviews by subspecialty if they continue virtually?

a. It would help a great deal as many residents still miss unpredictable time intermittently for interviews over the 4-month time period of interviews.
b. Would help, but not a major problem if virtual
c. If interviews continue virtually, staggering doesn't matter at all to me

In the space below please feel free to make comments or clarifications on any of your responses to the questions above

© 2022 Morgan HK et al. *JAMA Network Open.*
